# Calcitonin gene‐related peptide induces the histone H3 lysine 9 acetylation in astrocytes associated with neuroinflammation in rats with neuropathic pain

**DOI:** 10.1111/cns.13720

**Published:** 2021-08-16

**Authors:** Chenyan Sun, Qi An, Ruidi Li, Shuhui Chen, Xinpei Gu, Shuhong An, Zhaojin Wang

**Affiliations:** ^1^ Department of Human Anatomy Shandong First Medical University & Shandong Academy of Medical Sciences Taian China

**Keywords:** astrocytes, calcitonin gene‐related peptide, ChIP‐seq, chronic constriction injury, histone H3 lysine 9 acetylation, neuropathic pain

## Abstract

**Aims:**

Calcitonin gene‐related peptide (CGRP) as a regulator of astrocyte activation may facilitate spinal nociceptive processing. Histone H3 lysine 9 acetylation (H3K9ac) is considered an important regulator of cytokine and chemokine gene expression after peripheral nerve injury. In this study, we explored the relationship between CGRP and H3K9ac in the activation of astrocytes, and elucidated the underlying mechanisms in the pathogenesis of chronic neuropathic pain.

**Methods:**

Astroglial cells (C6) were treated with CGRP and differentially enrichments of H3K9ac on gene promoters were examined using ChIP‐seq. A chronic constriction injury (CCI) rat model was used to evaluate the role of CGRP on astrocyte activation and H3K9ac signaling in CCI‐induced neuropathic pain. Specific inhibitors were employed to delineate the involved signaling.

**Results:**

Intrathecal injection of CGRP and CCI increased the number of astrocytes displaying H3K9ac in the spinal dorsal horn of rats. Treatment of CGRP was able to up‐regulate H3K9ac and glial fibrillary acidic protein (GFAP) expression in astroglial cells. ChIP‐seq data indicated that CGRP significantly altered H3K9ac enrichments on gene promoters in astroglial cells following CGRP treatment, including 151 gaining H3K9ac and 111 losing this mark, which mostly enriched in proliferation, autophagy, and macrophage chemotaxis processes. qRT‐PCR verified expressions of representative candidate genes (ATG12, ATG4C, CX3CR1, MMP28, MTMR14, HMOX1, RET) and RTCA verified astrocyte proliferation. Additionally, CGRP treatment increased the expression of H3K9ac, CX3CR1, and IL‐1β in the spinal dorsal horn. CGRP antagonist and HAT inhibitor attenuated mechanical and thermal hyperalgesia in CCI rats. Such analgesic effects were concurrently associated with the reduced levels of H3K9ac, CX3CR1, and IL‐1β in the spinal dorsal horn of CCI rats.

**Conclusion:**

Our findings highly indicate that CGRP is associated with the development of neuropathic pain through astrocytes‐mediated neuroinflammatory responses via H3K9ac in spinal dorsa horn following nerve injury.

This study found that CGRP act on their astrocytic receptors and lead to H3K9 acetylation (H3K9ac), which are mainly associated with proliferation‐, autophagy‐, and inflammation‐related gene expression. The number of astrocytes with H3K9ac expression is increased after nerve injury. Inhibition of CGRP attenuates the development of neuropathic pain, which was accompanied by the suppression of H3K9ac, CX3CR1, and IL‐1β expression in CCI rats.

## Graphical abstract

This study find that CGRP act on their astrocytic receptors and lead to H3K9 acetylation (H3K9ac), which are mainly associated with proliferation‐, autophagy‐ and inflammation‐related gene expression. The number of astrocytes with H3K9ac expression is increased after nerve injury. Inhibition of CGRP attenuates the development of neuropathic pain, which was accompanied by the suppression of H3K9ac, CX3CR1, IL‐1β expression in CCI rats.

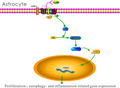

**Abbreviations**
AA: anacardic acid
BP: biological processes;
CCI: chronic constriction injury; CGRP, calcitonin gene‐related peptide
CGRP: calcitonin gene‐related peptide
ChIP‐seq: chromatin immunoprecipitation sequencing
CNS: central nervous system
CRCP: CGRP receptor component protein
CRLR: calcitonin receptor‐like receptor
GFAP: glial fibrillary acidic protein
GO: Gene Ontology
H3K9ac: histone H3 lysine 9 acetylation
HATs: histone acetyltransferases
HDACs: histone deacetylases
KEGG: Kyoto Encyclopedia of Genes and Genomes
MWT: mechanical withdrawal threshold
RAMP1: receptor activity‐modifying protein 1
RTCA: real‐time cell analysis
TWL: thermal withdrawal latency

## 1 INTRODUCTION

Astrocytes, the most abundant of glial cells, are critical for supplying nitrite to neurons and maintaining the homeostasis of the central nervous system (CNS). Increasing evidence suggests that astrocytes are involved in the persistent and development of chronic pain, as peripheral nerve injury of neuropathic pain models triggers astrocyte activation by induction of pro‐inflammatory mediator expression in astrocytes.^1^, ^2^ It has been reported that astrocyte activation is more closely associated with the development and maintenance of chronic neuropathic pain after nerve damage than microglial activation.^3^

Histone acetylation is a posttranslational modification that occurs at the N‐termini of the protein octamers and neutralizes the positive charge of lysine residues within histone tails.^4^ It is catalyzed through histone acetyltransferases (HATs) and is reversed via histone deacetylases (HDACs).^5^ The histone H3 lysine 9 acetylation (H3K9ac), a marker of actively transcribing genes, is associated with promoters and enhancers of actively transcribed genes.^6^ Increased H3K9ac enrichment regulated by HATs or HDACs in gene promoters is associated with transcriptional activation and increased gene expression.^7^, ^8^ There are a number of reports suggesting that H3K9ac enhances the transcription of cytokines and chemokines underlies the pathogenesis of chronic neuropathic pain after peripheral nerve injury.^9^, ^10^ Although some reports have shown that epigenetic mechanisms contribute to the development and maintenance of neuropathic pain,^11^, ^12^ further research is needed to clarify mechanisms regulating the expression of most key factors in this form of pain.

Calcitonin gene‐related peptide (CGRP) as an inflammation mediator has been proposed to contribute to nociceptive signaling through action on astrocytic CGRP receptors and release of ATP in dorsal horn.^13^ It has been reported that CGRP induced the activation of astrocytes at the transcriptional level through expression of the immediate‐early genes c‐fos and increased astrocytic marker of glial fibrillary acidic protein (GFAP) expression in spinal cord following peripheral nerve injury,^14^, ^15^, ^16^ suggesting that CGRP may play a physiological role as a regulator of astrocyte gene expression. Increased expression of GFAP represents astrocytic activation and gliosis following peripheral nerve injury.^17^ CBP/p300, one of the important histone acetyltransferase, can take part in the expression of GFAP gene through regulating H3K9 and H3K14 acetylation in the GFAP gene promoter and promote astrocyte differentiation in development.^18^, ^19^ Thus, we hypothesized that because CGRP regulates astrocytic activation following nerve injury, the inflammatory mediator may modify H3K9ac and H3K9ac‐mediated gene expression in these cells with participation in regulation of neuropathic pain.

Therefore, the present study was carried out to compare the different H3K9ac enrichments of astrocytes treated with CGRP and controls using chromatin immunoprecipitation sequencing (ChIP‐seq) to gain a better understanding of a potential role for this peptide in the activation of astrocytes. The effect of CGRP on the expression of H3K9ac in the spinal dorsal horn and the development of neuropathic pain were also examined in chronic constriction injury (CCI) rat model, hoping that these studies could further understand the underlying regulatory mechanism of astrocytes by CGRP in neuropathic pain pathophysiology.

## 2 MATERIALS AND METHODS

### 2.1 Animals and CCI rat model

Adult male Wistar rats weighing 200–250 g were obtained from the Animal Center of Shandong First Medical University. All animal experiments followed the guidelines of the Shandong First Medical University Institutional Animal Care and Use Committee and the ARRIVE guideline.^20^ CCI to the sciatic nerve of the right hind limb in rats was performed based on previous description.^21^ Briefly, animals were anesthetized with isoflurane (1.5%). The sciatic nerve of the right hind limb was exposed at the middle of the thigh by blunt dissection. To prevent the interruption of blood circulation through the epineurial vasculature, four chromic gut ligatures were loosely tied (4.0 silk) around the nerve with spacing at ~1 mm. In the control group, the right sciatic nerve was exposed for 2–3 min, but was not ligated. Following surgery, the skin was closed with a single suture. All behavioral tests were performed by mechanical withdrawal threshold (MWT) and thermal withdrawal latency (TWL) based on previous description.^21^ Mechanical allodynia and thermal hyperalgesia are reproducible and sensitive behavioral readouts of neuropathic pain.

### 2.2 Intrathecal implantation

Intrathecal implantation was performed as described previously^22^, ^23^ by inserting polyethylene tubing through which the drug was directly injected into the subarachnoid space of the lumbar enlargement. After surgery, neurologically normal rats were injected with 2% lidocaine (10 μl) through the intrathecal catheter to confirm that the polyethylene tubing was in the subarachnoid space. Only those rats showing complete paralysis of both hind limbs after the administration of lidocaine were used for subsequent experiments. Animals with the intrathecal catheter were then randomly divided into CCI and sham operation, respectively. The CGRP (1 μM, Tocris Bioscience), anacardic acid (AA, HAT inhibitor, 20 μM, Abcam), CGRP8‐37 (CGRP antagonist, 2 μM, MCE), or vehicle in a volume of 10 μl was injected into the spinal lumbar enlargement region through the intrathecal catheter, followed by 20 μl of saline to flush. Previous studies have demonstrated that these dosages of CGRP, AA, CGRP8‐37 and other reagents in experiments proved to be effective in vivo and in vitro.^13^, ^24^, ^25^ When the drug administration fell on same day as the behavior analysis, behavior tests were completed prior to the drug administration. At the end of each experiment, the position of the polyethylene tubing in the intrathecal space at the lumbar enlargement was visually verified by exposing the lumbar spinal cord. Data from rats with incorrect polyethylene tubing position were discarded from the study. The experimenter who conducted the behavioral tests was blinded to treatments given to the animals.

### 2.3 Cell culture and drug administration

Rat C6 astroglial cells, those are positive for differentiated astrocytic markers, (e.g., GFAP, S100B, AQP4, ALDH1L1)^26^ were obtained from the Cell Bank of the Chinese Academy of Sciences (Beijing, China). Cells were cultured in DMEM supplemented with 10% fetal bovine serum (FBS, Biological Industries) incubated at 37℃ in an atmosphere of 5% CO_2_. Astrocytic cells continuously stimulated with CGRP peptide (1 μM) at 0, 1, 2, 4, 6, and 12 h, respectively. Cells without CGRP peptide were used as control. To assess the possible underlying HATs for the effect of CGRP, 20 μM AA was pre‐applied for 30 min and co‐applied together with CGRP for 4 h at 37℃.

### 2.4 Immunofluorescence of spinal cord

Animals were perfused through the ascending aorta with 100–150 ml saline followed by 300 ml 4% paraformaldehyde in 0.1 M phosphate buffer (pH 7.4). L4‐L5 spinal cord segments were removed, post‐fixed in the same fixative for 4 h at 4℃, and cryoprotected in 30% sucrose overnight. Transverse 8‐μm‐thick sections were cut on a cryostat and processed for immunofluorescence. In order to reveal the coexistence of H3K9ac with GFAP (a marker for astrocytes), Iba1 (a marker for microglia) or NeuN (a marker for neurons), and GFAP with CGRP, respectively, double immunostaining on the same section was used. Sections were incubated with primary antibodies against H3K9ac (Abcam) with GFAP (Abcam), Iba1 (Abcam) or NeuN (Abcam), and GFAP with CGRP (Millipore) overnight at room temperature. Following three washes with tris‐buffered saline (TBS), sections were treated with a 1:1 mixture of the matching FITC and Cy3‐conjugated secondary antibodies (Jackson Immunoresearch). After washing three times in TBS, sections were counterstained with DAPI (Abcam). The specificity of antibodies used was checked by Western blotting and/or omission of the primary antibodies. No immunoreactive products were detected in these tissue sections after omitting primary antibodies.

### 2.5 Quantification of immunofluorescence

Quantitative analysis of the percentage of immunostaining surface in the spinal cord laminae I‐II (CGRP) and whole spinal dorsal horn (laminae I‐VI, GFAP) was conducted with Image Pro‐Plus program as described previously.^23^ Briefly, the background in pictures was first subtracted with a uniform standard. The regions for laminae I‐II and whole spinal dorsal horn in the spinal sections were artificially selected. Then, threshold values of fluorescent intensity for positive immunoreactivity were set and the percentage of immunostaining areas were obtained by the Image Pro‐Plus program 6.0. The percentage of H3K9ac/GFAP, H3K9ac/Iba1, and H3K9ac/NeuN double‐labeled cells relative to the total number of GFAP, Iba1, and NeuN‐positive cells in the L4‐5 spinal dorsal horn visualized per section were calculated and averaged, respectively.

### 2.6 Immunofluorescence of cultured astroglial cells

Rat astroglial cells (C6) were cultured on poly‐l‐lysine‐coated coverslips. Following a single wash in phosphate buffered saline (PBS), cultured astroglial cells were fixed in 4% paraformaldehyde for 15 min at room temperature. Double‐labeling immunofluorescence staining for primary antibodies against GFAP and calcitonin receptor‐like receptor (CRLR; Abcam), receptor activity‐modifying protein 1 (RAMP1; Sigma Aldrich), or CGRP receptor component protein (CRCP, Proteintech) on coverslip cultured astroglial cells was performed. Coverslips were incubated with a mixture of the two primary antibodies overnight. Coverslips were then incubated with FITC and Cy3‐conjugated secondary antibodies. After washing three times in TBS, coverslips were counterstained with DAPI.

### 2.7 Western blotting

Cultured astroglial cells or the dorsal quadrant of L4‐L5 spinal segment ipsilateral to the operation side were lysed, and the protein was extracted. The protein lysate from each sample was separated electrophoretically on a sodium dodecyl sulfate‐polyacrylamide gel and then transferred to a polyvinylidene fluoride (PVDF) membrane. After blocking with 5% nonfat milk in TBS‐T (containing 0.1% Tween‐20) for 2 h, membranes were incubated with primary antibodies against CGRP, GFAP, H3K9ac, CX3CR1 (CST), LC3B (Abcam), and IL‐1β (Abcam) in 5% nonfat milk in TBS‐T overnight at 4℃. After washing with TBS‐T, membranes were incubated with the appropriate secondary antibodies for 2 h. Results were visualized using an ECL chemiluminescence system. GAPDH antibody (CST) was also used as a probed control to ensure the loading of equivalent amounts of the sample proteins. The band densities were compared in TotalLab software (version 2.01; Bio‐Rad, Hercules, CA).

### 2.8 Real‐time cell analysis (RTCA)

Astroglial cells were seeded at 10^3^ cells/well in 96‐well E‐plates (Roche) with an integrated microelectronic sensor array in 100 μl of suitable culture medium (RTCA DP; ACEA Biosciences). After 24 h, 1 μM CGRP or 1 μM CGRP with either 20 μM AA or 2 μM CGRP8‐37 was added to a total volume of 100 μl. Cell proliferation and viability were monitored in real‐time by measuring the cell‐to‐electrode responses of seeded cells. The cell index (CI) was calculated for each E‐plate well by RTCA Software. The graph is generated in real‐time by the xCELLigence system.

### 2.9 Chromatin immunoprecipitation (ChIP)

Chromatin was prepared from fixed rat astroglial cells (stimulated with 1 μM CGRP, 4 h) and sonicated fragments ranged in size from 200 to 1500 bp. Approximately 2 × 10^7^ cell equivalents were used for each immunoprecipitation. ChIP was performed as described previously,^27^ using anti‐H3K9ac antibody (ChIP Grade, ab10812, Abcam) or a control rabbit IgG.

### 2.10 Sequencing library preparation, cluster generation, and sequencing

DNA samples were end‐repaired, A‐tailed, and adaptor‐ligated using TruSeq Nano DNA Sample Prep Kit (#FC‐121‐4002, Illumina), following manufacturer's instructions. Approximately200‐1500 bp fragments were size selected using AMPure XP beads. The final size of the library was confirmed by Agilent 2100 Bioanalyzer. Samples were diluted to a final concentration of 8 pmol/L and cluster generation was performed on the Illumina cBot using HiSeq 3000/4000 PE Cluster Kit (#PE‐410‐1001, Illumina), following manufacturer's instructions. Sequencing was performed on Illumina HiSeq 4000 using HiSeq 3000/4000 SBS Kit (300 cycles) (#FC‐410–1003, Illumina), according to the manufacturer's instructions.

### 2.11 Data collection and ChIP‐seq analysis

After the sequencing platform generated sequencing images, stages of image analysis and base calling were performed using Off‐Line Basecaller software (OLB V1.8). Sequence quality was examined using the FastQC software. After passing Solexa CHASTITY quality filter, clean reads were aligned to rat genome (UCSC RN6) using BOWTIE software (V2.1.0). Aligned reads were used for peak calling of the ChIP regions using MACS V1.4.2. Statistically significant ChIP‐enriched regions (peaks) were identified by comparison of IP vs Input or comparison to a Poisson background model, using a *p*‐value threshold of 10^−4^. Peaks in samples were annotated by the nearest gene using the newest UCSC RefSeq database. The annotation of the peaks which were located within −2Kb to +2Kb around the corresponding gene of transcription start site (TSS) in samples can be found from the peaks‐promoter‐annotation.

### 2.12 Bioinformatics analysis

The Gene Ontology (GO) functional and Kyoto Encyclopedia of Genes and Genomes (KEGG) pathway enrichment analyses were performed using the Database for Annotation, Visualization, and Integrated Discovery (DAVID) and KEGG Orthology‐Based Annotation System (KOBAS) online tools (http://www.geneontology. org and http://www.genome.jp/kegg).

### 2.13 RNA extraction and quantitative real‐time PCR (qRT‐PCR)

The expression profiles of genes selected from enriched GO terms that derived from ChIP‐seq data were assessed by qRT‐PCR at 4 h after treatment of CGRP with astroglial cells. The expression of GAPDH mRNA was also determined as an internal control. Total RNA was isolated from cultured astroglial cells using Trizol reagent (Invitrogen) according to the manufacturer's protocol. RNA concentration was determined spectrophotometrically. After this, cDNA was synthesized using a cDNA synthesis kit (Invitrogen) according to the manufacturer's instructions. Primer sequences are listed in the Table 1. qRT‐PCR was performed in triplicates by using a 7300 real‐time PCR system (Applied Biosystems, Foster City, CA) according to the manufacturer's instructions. A comparative cycle of threshold fluorescence (ΔCt) method was used, and the relative transcript amount of target gene was normalized to that of GAPDH using the 2^−ΔΔCt^ method. The final results of qRT‐PCR were expressed as the ratio of test mRNA to control.

**TABLE 1:** Primers used for real‐time quantitative PCR

Gene	Primer sequence (5′ to 3′)	Annealing temperature (℃)	Size (bp)
GAPDH	F:5′ GCTCTCTGCTCCTCCCTGTTCTA3′	60	124
	R:5′ TGGTAACCAGGCGTCCGATA3′		
ATG12	F:5′ AGAAACAGCCATCCCAGAGC3′	60	147
	R:5′ TCCACAGCCCATTTCTTCGT3′		
ATG4C	F:5′ ACCTCCAACACCATCCACAA3′	60	105
	R:5′ CTTCCCGGACGTTTCCTTC3′		
Hmox1	F:5′ GGTCCTGAAGAAGATTGCG3′	60	258
	R:5′ GAGGGACTCTGGTCTTTGTG3′		
Ret	F:5′ GCTGTCCCGAGATGTTTATGAA3′	60	140
	R:5′ GCAGCACTCCAAAGGACCAC3′		
Cx3cr1	F:5′ CCTTTGGGACCATCTTCCTATC3′	60	70
	R:5′ AACAGATTCCCCACCAGACC3′		
Mtmr14	F:5′ CTCTGACGACCGCCTACCTT3′	60	209
	R:5′ GAATACTGCCACAGCCCGATA3′		
MMP28	F:5′ GCAGAACCTGTACGGAAAGC3′	60	157
	R:5′ GATGGCATCAAAGGAAGAGTG3′		

### 2.14 Statistical Analysis

The data were analyzed using GraphPad Prism, version 6.0 (GraphPad Software, Inc, San Diego, CA). Data are presented as the means ± SEM. Shapiro‐Wilk tests were used to evaluate the normality of the distribution. Mann‐Whitney U‐tests were used for comparisons between two groups, and Kruskal‐Wallis tests with Dunn's multiple comparisons post hoc tests were used for comparisons among multiple groups. The MWT or TWL among groups was analyzed by two‐way repeated measures ANOVA with groups and time points as independent factors, followed by Bonferroni post hoc tests. Significance was set at *p* < 0.05.

## 3 RESULTS

### 3.1 Model identification of neuropathic pain

To assess the chronic pain behavior induced by CCI in rat model, both mechanical allodynia and thermal sensitivity of animal hind paws were evaluated on postoperative days 0, 1, 3, 5, 7, 10, and 14, respectively (Figure S1). MWT and TWLs of CCI‐ipsilateral hind paws were significantly lower than those of both sham‐ipsilateral at days 3–14 after CCI operation and reached a steady peak on postoperative day 14, suggesting the CCI‐induced mechanical allodynia and thermal hyperalgesia of hind paws.

### 3.2 CCI evoked increases in the expression of CGRP and GFAP in spinal dorsal horn

**FIGURE 1:**
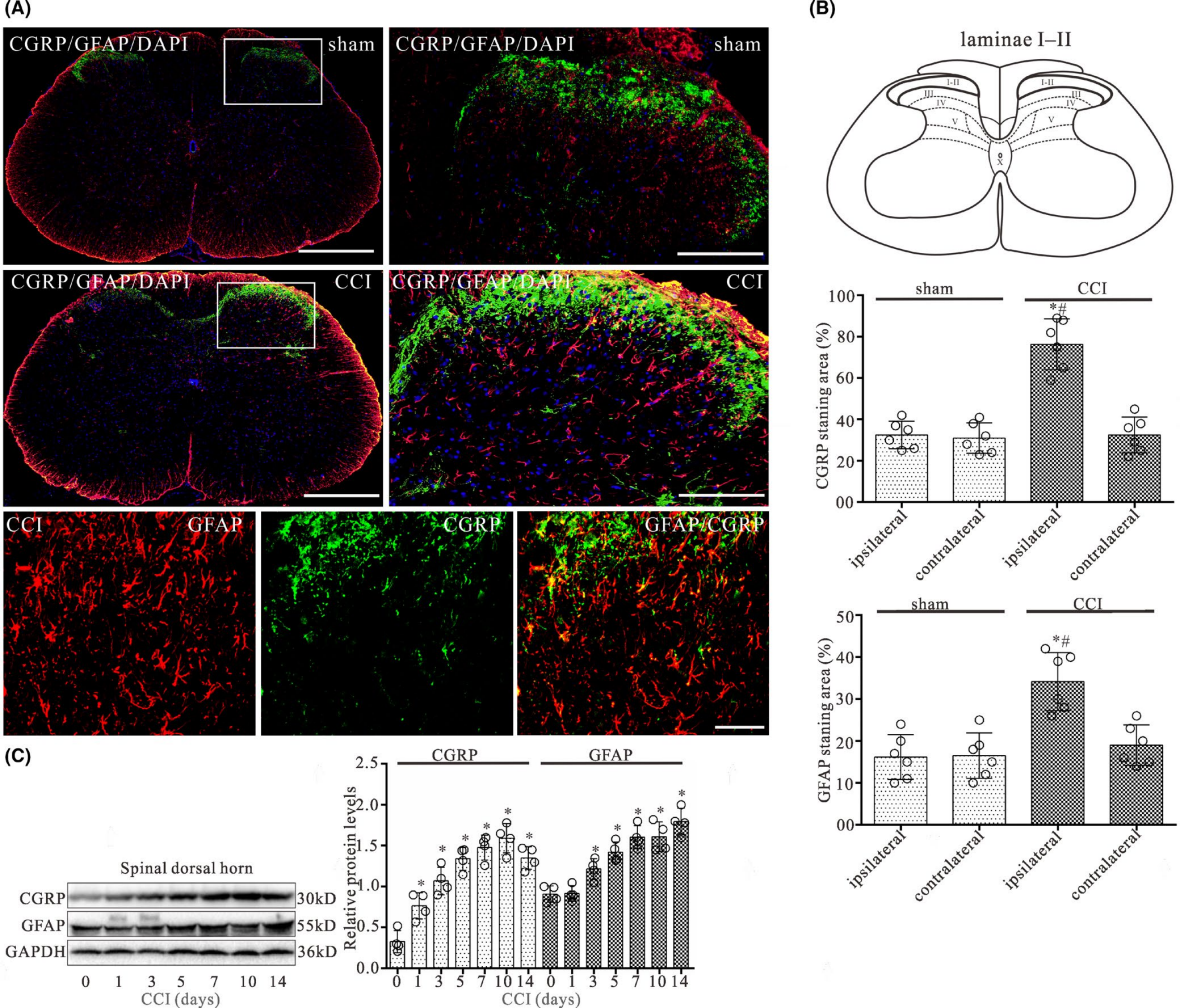
CCI evokes increases in the expression of CGRP and GFAP in the L4‐L5 spinal dorsal horn of CCI rats. (A) Double‐staining immunofluorescent images showing CGRP‐positive fibers (green) and GFAP (astrocyte maker)‐positive astrocytes (red) in the dorsal horn of sham and CCI groups on day 14 after surgery. The second column of first two rows is the higher magnification images indicated in the white boxes in the first column of first two rows. Cell nuclei were stained with the DAPI (blue). Note that numerous varicose nerve terminals immunoreactive for CGRP (green) closely approached GFAP‐immunopositive astrocytes (red) in the laminae I and II of spinal dorsal horn (third row). Scale bar 500 μm in the first column and 200 μm in the second column of the first two rows, 10 μm in third row. (B) Quantitative analyses of the percentages of CGRP‐immunoreactive surface in laminae I and II and GFAP‐immunostaining surface in spinal dorsal horn showed the CCI‐induced changes. Data are presented as the mean ± SEM (*n* = 6). *p < 0.05 versus sham‐ipsilateral; #*p* < 0.05 versus CCI‐contralateral. (C) Western blot analyses of CGRP and GFAP expression in the dorsal quadrant of L4‐L5 spinal segment ipsilateral to the operation side on 0, 1, 3, 5, 7, 10, and 14 day after CCI surgery, respectively. The mean optic densities of the protein were calculated by normalizing to GAPDH. All values are expressed as the means ± SEMs (*n* = 4).*p < 0.05 versus sham group

CGRP immunoreactivity in laminae I‐II and GFAP immunoreactivity in the whole dorsal horn of the L4‐L5 spinal cord did not differ between the operated and unoperated sides in the sham control (Figure 1A). On postoperative day 14, the staining surface percentages of CGRP in laminae I‐II and GFAP in the whole dorsal horn ipsilateral to CCI were increased relative to the sham‐operated group and the contralateral side (*p* < 0.05) (Figure 1A, B). Double immunofluorescence revealed that numerous varicose nerve terminals immunoreactive for CGRP closely approached GFAP‐immunopositive astrocytes in the ipsilateral spinal dorsal horn (Figure 1A). However, GFAP‐immunoreactive astrocytes never expressed CGRP immunoreactivity.

Western blot data showed that CCI evoked significant increases in both CGRP and GFAP protein expressions of the dorsal quadrant of L4‐L5 spinal segment ipsilateral to the operation side on postoperative days 3, 5, 7, 10, and 14, respectively (Figure 1C). The largest increases in expression for these time points were seen on day 10 for CGRP and on day 14 for GFAP post‐operation.

### 3.3 CCI induced increase of H3K9ac expression in the spinal dorsal horn

**FIGURE 2:**
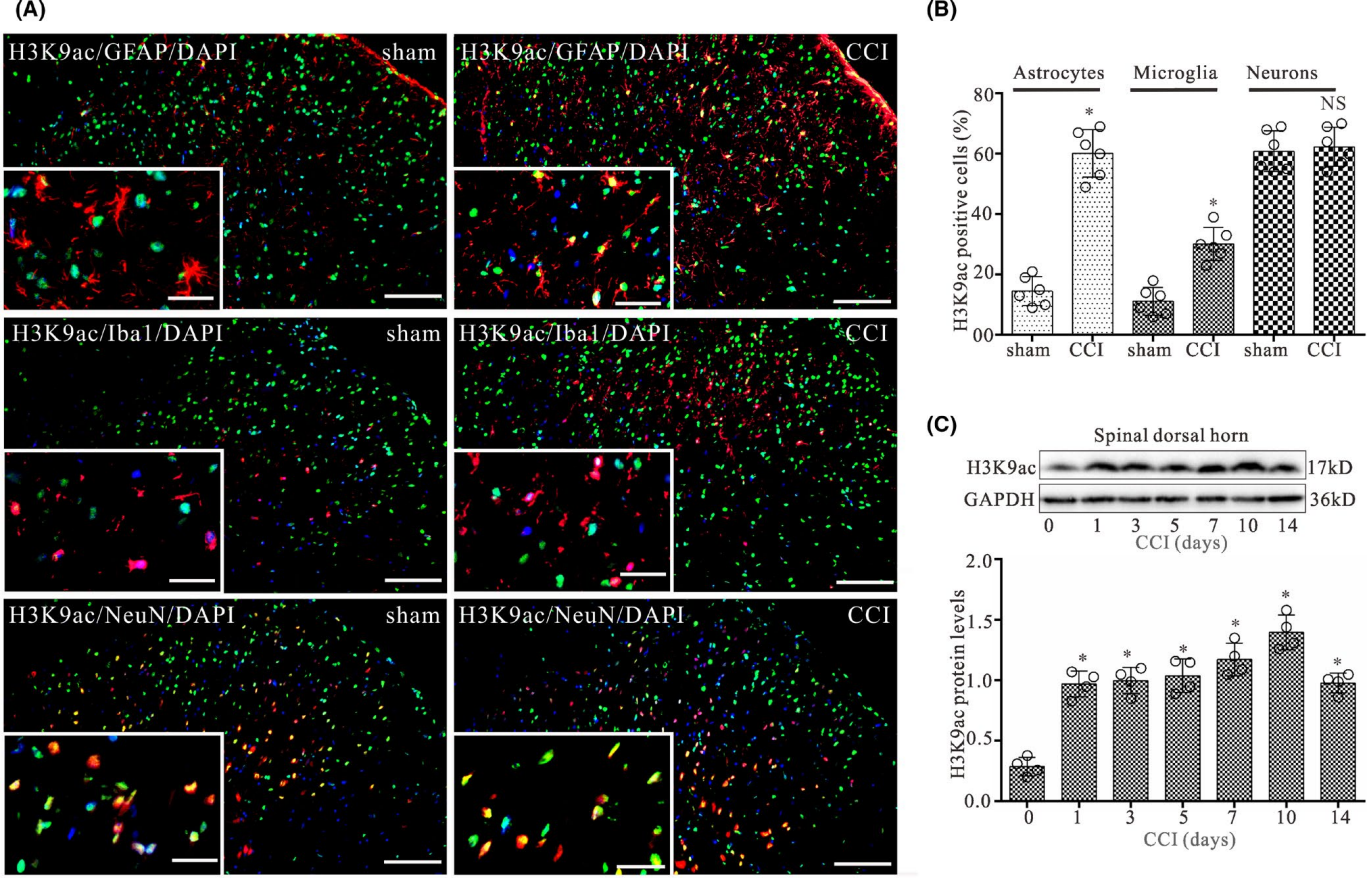
CCI evokes increases in glial cells displaying H3K9ac expression in the spinal dorsal horn on day 14 after surgery. (A) Double‐staining immunofluorescent images showing the distribution of H3K9ac (green) in astrocytes (GFAP, red), microglia (Iba1, red), and neurons (NeuN, red) in L4‐L5 spinal dorsal horn of CCI and control groups on day 14 after surgery. Cell nuclei were stained with the DAPI (blue). Images in the white boxes are the amplification of an area in the corresponding image. Scale bar 100 μm outside the white frame and 25 μm in the white frame. (B) Graphs showing the percentage of GFAP/H3K9ac, Iba1/H3K9ac, and NeuN/H3K9ac double‐labeled cells relative to the total number of GFAP, Iba1, and NeuN‐positive cells in L4‐L5 spinal dorsal horn ipsilateral to the operation side on day 14 after CCI surgery, respectively. Data are presented as the mean ± SEM (*n* = 6). *p < 0.05. NS, no statistical difference. (C) Western blot analysis of H3K9ac expression in the dorsal quadrant of L4‐L5 spinal segment ipsilateral to the operation side on days 0, 1, 3, 5, 7, 10, and 14 after CCI surgery, respectively. The mean optic densities of the protein were calculated by normalizing to GAPDH. All values are expressed as the means ± SEMs (*n* = 4).*p < 0.05 versus sham

Immunofluorescent double staining showed the specific cell type of the changes for H3K9ac expression in the spinal dorsal horn of CCI rat. In sham rats, H3K9ac is mainly expressed in neurons and a few in astrocytes or microglia in the spinal dorsal horn (Figure 2A). However, 14 days after CCI surgery, H3K9ac was sharply increased in astrocytes, and slightly increased in microglia, with little to no change in neurons in the spinal dorsal horn (Figure 2A, B).

Western blot data showed that CCI evoked significant increase in H3K9ac protein expression of the dorsal quadrant of L4‐L5 spinal segment ipsilateral to the operation side on postoperative days 1, 3, 5, 7, 10, and 14, respectively (Figure 2C). The largest increase in expression for these time points was seen at 10 days post‐operation.

### 3.4 CGRP8‐37 and HAT inhibitor prevented the development of neuropathic pain

**FIGURE 3:**
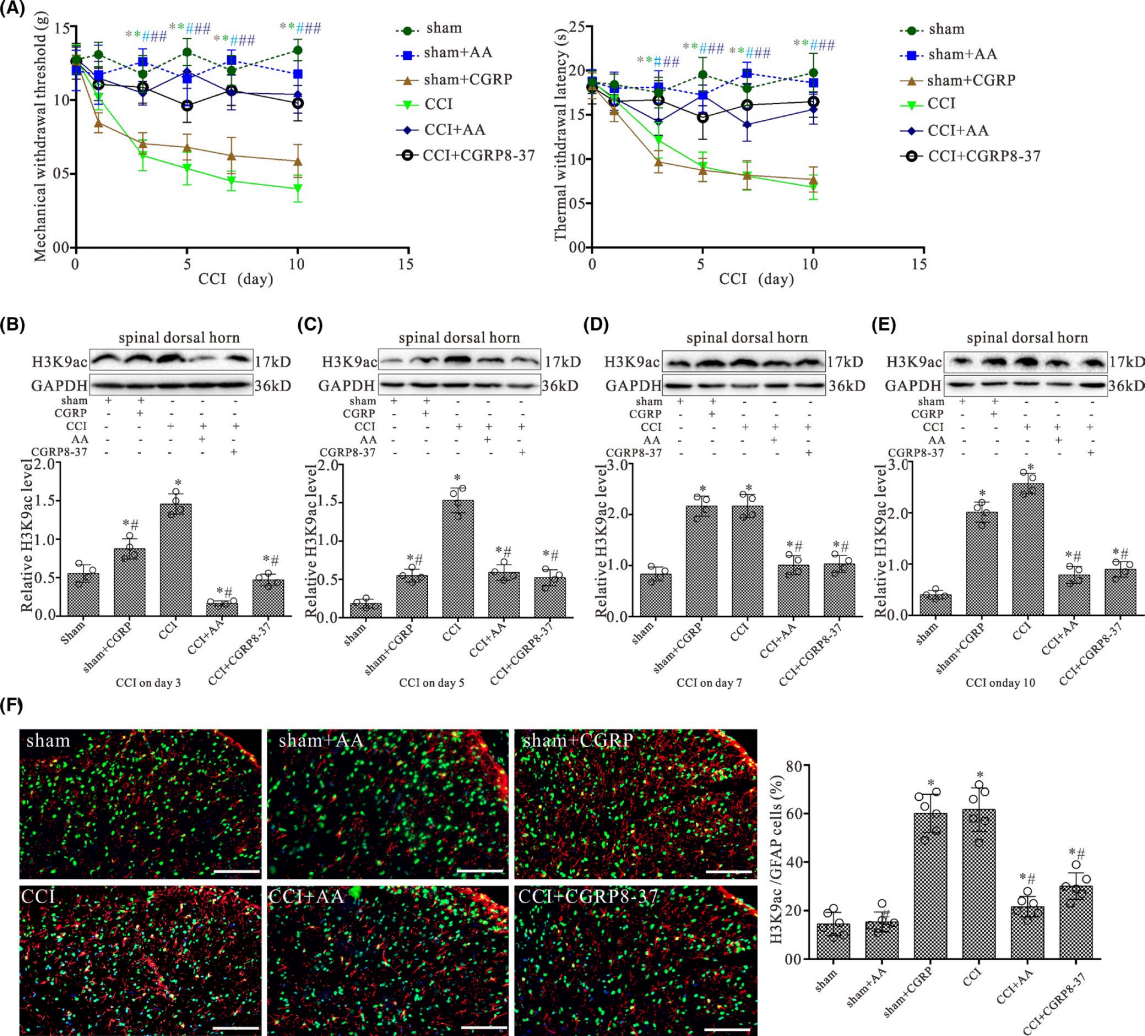
Intrathecal CGRP antagonist and HAT inhibitor administration prevent the pain hypersensitivity and attenuate increased levels of H3K9ac in the spinal dorsal horn induced by CCI. (A) Shows the mechanical withdrawal threshold (MWT) and thermal withdrawal latency (TWL) during the 10‐day observation period in rats treated with daily intrathecal injection of either 1 μM CGRP, 20 μM AA (HAT inhibitor), 2 μM CGRP8‐37, or vehicle in 10 μl for 9 days. All values are expressed as the means ± SEMs (*n* = 6). (B–E) Western blot analyses for H3K9ac protein levels in the dorsal quadrant of L4‐L5 spinal segment ipsilateral to the operation side with CCI surgery for 3, 5, 7, and 10 days, respectively. Data were obtained from animals treated with daily intrathecal injection of either 1 μM CGRP, 20 μM AA, 2 μM CGRP8‐37, or vehicle in 10 μl for 2, 4, 6, and 9 days, respectively. The mean optic densities of the proteins were calculated by normalizing to GAPDH. All values are expressed as the means ± SEMs (*n* = 4).*p < 0.05 versus sham groups; #*p* < 0.05 versus CCI alone groups. (F) Double‐staining immunofluorescent images showing the expression of H3K9ac (green) in astrocytes (GFAP, red) of L4‐L5 spinal dorsal horn ipsilateral to the operation side in the sham, sham + AA, sham + CGRP, CCI, CCI + AA, and CCI + CGRP8‐37 groups on day 10 after surgery, respectively. Scale bar 100 μm. Graph showing the percentages of GFAP/H3K9ac double‐labeled cells in L4‐L5 spinal dorsal horn of the sham, sham + AA, sham + CGRP, CCI, CCI + AA, and CCI + CGRP8‐37 groups, respectively. Data are presented as the mean ± SEM (*n* = 6). *p < 0.05 versus sham groups; #*p* < 0.05 versus CCI alone groups

To determine the effect of CGRP on the development of chronic pain via H3K9ac in CCI rats, we examined whether CGRP8‐37 (CGRP antagonist) and AA (HAT inhibitor) can prevent the development of neuropathic pain. Rats were randomly assigned into six groups: sham, sham + AA, sham + CGRP, CCI, CCI + AA, and CCI + CGRP8‐37 groups. Behavior analyses were performed on day 1 before the surgery and then on postoperative days 1, 3, 5, 7, and 10, respectively. Rats in the sham + AA, sham + CGRP, CCI + AA, and CCI + CGRP8‐37 groups received either CGRP (1 μM), AA (20 μM) or CGRP8‐37 (2 μM) in 10 μl through the pre‐implanted intrathecal catheter on day 1 immediately prior to the surgery and then daily till day 9 after the surgery. Vehicle (10 μl) were administered to rats in the CCI and sham groups as controls. As shown in Figure 3A, MWT and TWL in CCI and sham + CGRP groups were significantly lower than those of both in sham groups on postoperative days 3–10 (*p* < 0.05; *n* = 6). Compared with CCI alone, MWT and TWL in CCI + AA and CCI + CGRP8‐37 groups were significantly higher than those of both in CCI group on postoperative days 3–10 (*p* < 0.05; *n* = 6). However, the MWT and TWL did not significantly differ between sham + AA and sham alone.

### 3.5 CGRP8‐37 and HATs inhibitor inhibited the increase of H3K9ac by CCI in the spinal dorsal horn

H3K9ac protein level in the dorsal quadrant of L4‐L5 spinal segment ipsilateral to the operation side was examined following treatment of AA and CGRP8‐37 with CCI rats by Western blot analysis. Animal grouping and treatment of CGRP, AA, and CGRP8‐37 are the same as the animal behavioral tests described above. As shown in Figure 3B–E, CCI and CGRP treatment significantly increased in H3K9ac protein level on postoperative days 3, 5, 7, and 10, respectively (*p* < 0.05; *n* = 4). Compared with CCI alone, CCI with AA and CGRP8‐37 markedly reversed the CCI induced the increase of H3K9ac protein expression (Figure 3B–E).

H3K9ac and GFAP immunostaining in animal receiving CGRP intrathecally as well as AA and CGRP8‐37 were also examined. Intrathecal administration of CGRP and CCI significantly increased the percentage of H3K9ac/GFAP double‐labeled astrocytes in the spinal dorsal horn of rats (Figure 3F, *p* < 0.05; *n* = 6). Intrathecal administration of AA and CGRP8‐37 can prevent the increase of H3K9ac/GFAP double‐labeled astrocytes in the spinal dorsal horn of CCI rats (*p* < 0.05; *n* = 6). However, H3K9ac/GFAP double‐labeled astrocytes in the spinal dorsal horn did not differ between sham + AA group and sham alone.

### 3.6 CGRP increased H3K9ac expression in astroglial cells by HATs

**FIGURE 4:**
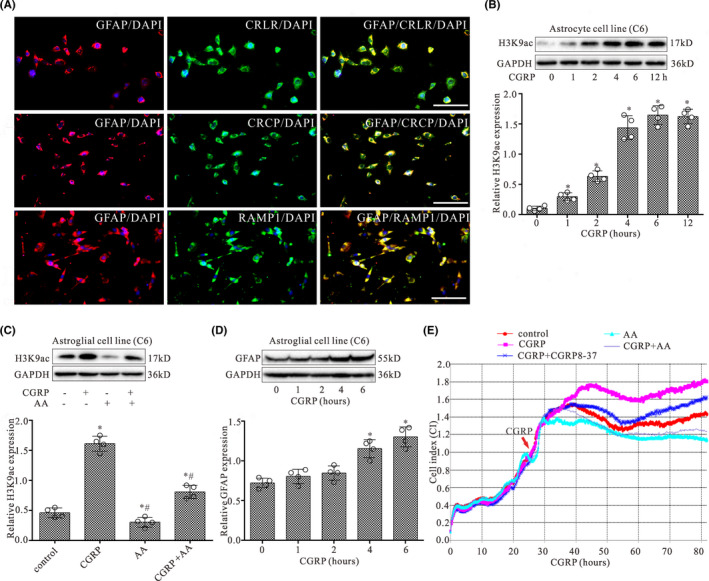
CGRP evokes increases in the expression of H3K9ac in astroglial cells by HATs. (A) The expressions of GFAP (a marker of astrocytes, red) and its co‐localization with CRLR (green), RAMP1 (green), or CRCP (green) staining in cultured astroglial cells (C6). Scale bar 40 μm. (B) Western blot analysis of H3K9ac expression in astrocytic cell line (C6) with treatment of CGRP at 0, 1, 2, 4, 6, and 12 h, respectively. (C) Western blot analyses for H3K9ac protein levels in astroglial cells (C6) with co‐treatment of CGRP (1 μM) and AA (20 μM) for 4 h. (D) Western blot analysis of GFAP expression in astroglial cell line (C6) with treatment of CGRP at 0, 1, 2, 4, and 6 h, respectively. The mean optic densities of the proteins were calculated by normalizing to GAPDH. All values are expressed as the means ± SEMs (*n* = 4).*p < 0.05 versus controls; #*p* < 0.05 versus CGRP only groups. (E) Shown is an example of astrocytic cell growth curves by RTCA. RTCA was performed to evaluate the proliferation and viability of astroglial cells with continuous treatment of CGRP and co‐treatment of AA or CGRP8‐37

To study the effect of CGRP on astrocytes, we first investigated the expression of CGRP receptor components on astrocytes. Figure 4A shows an example of co‐localization of CRLR, RAMP1, and CRCP with the GFAP immunoreactivity on astroglial cells in culture. Nearly all of the GFAP‐positive cells expressed CGRP receptor components CRLR, RAMP1, and CRCP.

The expression of H3K9ac in astroglial cells was assessed by Western blot following treatment with CGRP for 0, 1, 2, 4, 6, and 12 h, respectively. As shown in Figure 4B, CGRP treatment significantly increased the H3K9ac protein level in astroglial cells in a time‐dependent manner with a maximal effect observed after 6 h. However, CGRP with AA (HATs inhibitor) partially or completely blocked the increased effect of CGRP on the expression of H3K9ac (Figure 4C). Furthermore, CGRP treatment significantly increased the GFAP protein level in astroglial cells after CGRP treatment for 4 and 6 h (Figure 4D).

### 3.7 CGRP promoted the proliferation and viability of astroglial cells

In order to determine the effect of CGRP on astroglial cells, the cell proliferation and viability were assessed using RTCA following treatment of astroglial cells with CGRP. RTCA proliferation assay demonstrated that the cell index increased in a time‐dependent manner following CGRP treatment and was significantly higher in CGRP group when compared with the control group following treatment after 20‐h treatment (Figure 4E). Compared with CGRP alone, CGRP with AA or CGRP8‐37 partially or completely blocked the increased effect of CGRP on cell proliferation and viability after 20‐h treatment (*p* < 0.05; *n* = 3).

### 3.8 Genome‐wide profile of H3K9ac targets in astroglial cells after CGRP treatment

**FIGURE 5:**
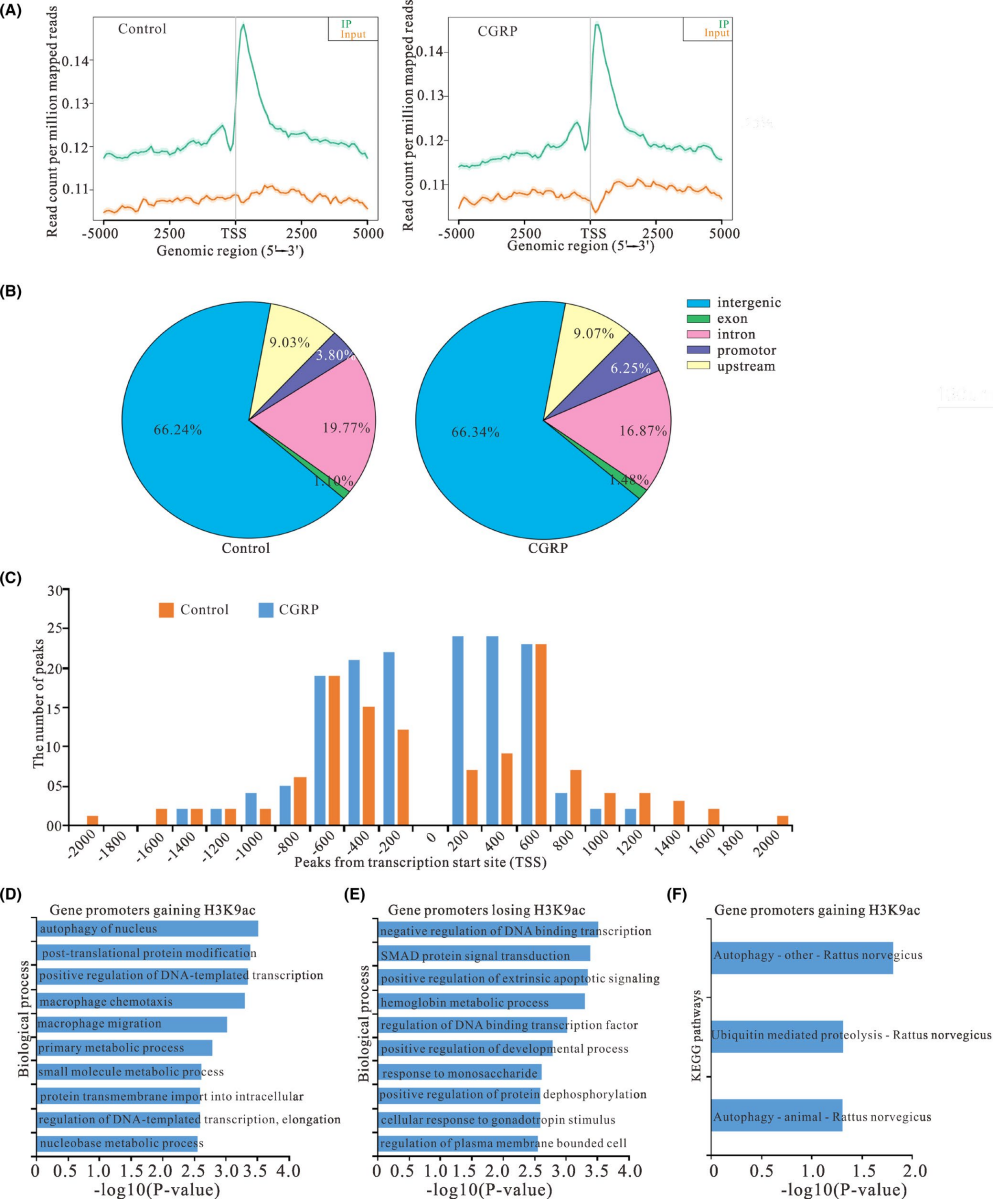
Effect of CGRP on the peak distribution of the ChIP‐seq reads of H3K9ac in astroglial cells treated with CGRP compared with control. (A) Metagenes representation of average enrichment profiles of H3K9ac in astroglial cells treated with CGRP and control. The y axis represents the numbers of the total sites that were identified as H3K9ac peaks. (B) The distribution of CGRP‐mediated H3K9ac peaks relative to annotated genes in astroglial cells treated with CGRP and control. (C) The distribution of H3K9ac enrichment peaks on promoters relative to gene transcription start sites (TSSs). Shown is H3K9ac peak frequencies relative to the distance from the nearest annotated TSS in astroglial cells treated with CGRP and control. (D, E) GO annotation of genes gaining H3K9ac (D) and genes losing this mark (E) of CGRP treatment group versus control. Bar plots show the top 10 enrichment values of the significant enrichment terms involving biological process (BP). (F) KEGG pathway analysis of genes gaining H3K9ac in astroglial cells treatment with CGRP. The bar plots show enrichment values of the significant enrichment terms involving KEGG pathways

The distribution of H3K9ac enrichments on gene promoters was examined in CGRP‐treated astroglial cells and controls. Average H3K9ac profiles are similar in control and CGRP‐treated cells (Figure 5A). A strong enrichment of H3Kac occurs from −2000 to +2000 bp across TSSs, including many sites located in downstream proximal regions of TSSs in CGRP‐treated astroglial cells or controls (Figure 5A), corresponding to the position of the nucleosome‐depleted zone.^28^ However, the proportions of intergenic, exon, intron, promoter, and upstream were different in CGRP‐treated cells, compared to controls (Figure 5B). In addition, we identified a total of 262 gene promoters, whose H3K9ac enrichments are significantly altered in astroglial cells treated with CGRP, including 151 gaining H3K9ac, and 111 losing this mark, compared with controls (Table S1). The distribution of H3K9ac‐enriched promoters was mapped to proximal regions of TSSs of RefSeq genes (Figure 5C).

### 3.9 GO analysis of peaks relative to annotated genes

According to the functional annotation in GO database, gene promoters gaining H3K9ac were mostly enriched for biological processes (BP) terms associated with autophagy of nucleus (ATG4C, ATG12), positive regulation of DNA‐templated transcription (SUPT4H1, ALYREF), and macrophage chemotaxis (MMP28, CX3CR1) (Figure 5D; Table S2), which are closely associated with the activation of astrocytes in neuropathic pain.

Meanwhile, gene promoters losing H3K9ac were enriched in BP terms, such as negative regulation of DNA‐binding transcription factor activity (CAT, GFI1, HMOX1, HNF4A) and positive regulation of extrinsic apoptotic signaling pathway (RET, INHBA) (Figure 5E; Table S3).

### 3.10 KEGG pathway analysis of peaks relative to annotated genes

Based on the KEGG pathway enrichment analysis, gene promoters gaining H3K9ac were significantly enriched in three signaling pathways, including both autophagy pathways (ATG12, ATG4C, MTMR14) (Figure 5F; Table S4), which were mostly related to mediation of neuropathic pain induced by CCI. However, none of gene promoters losing H3K9ac was significantly enriched in any KEGG pathway.

### 3.11 CGRP altered the gene expression in astroglial cells associated with neuroinflammation

**FIGURE 6:**
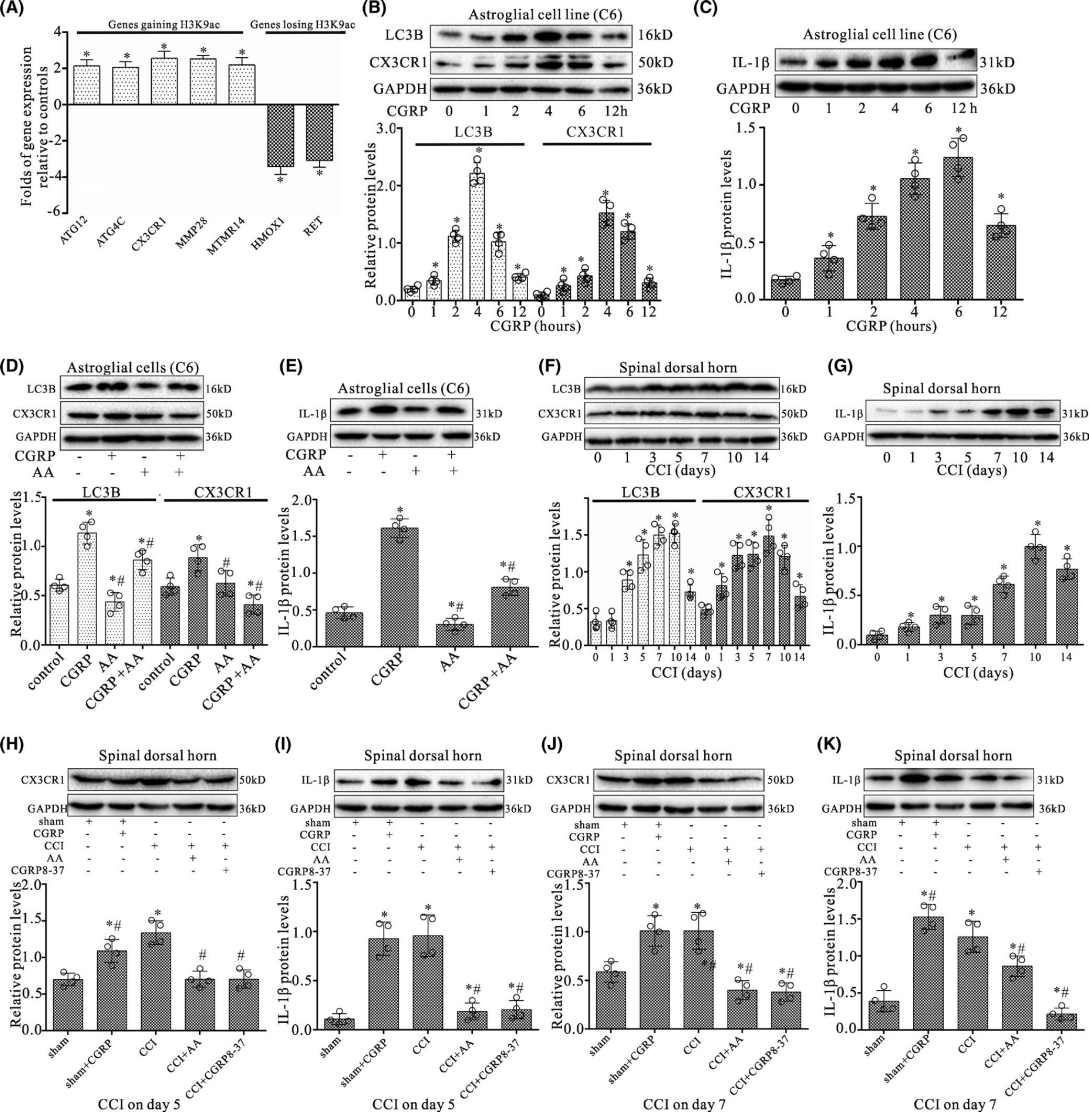
CGRP altered the gene expression in astroglial cells associated with astrocyte activation. (A) Quantitative RT‐PCR analysis for differences in expression levels of H3K9ac specific target genes between CGRP‐treated astroglial cells and controls in the subset of genes gaining or losing H3K9ac on their promoters. Results were calculated by normalizing to GAPDH in the same sample with the ΔCt method. Changes in relative levels of gene mRNAs expressed as folds of controls. All values were mean ± SEM. *p < 0.05 (*n* = 3). (B, C) Western blot analyses of CX3CR1 and LC3B (B) or IL‐1β (C) expressions in astroglial cells (C6) with treatment of CGRP at 0, 1, 2, 4, 6, and 12 h, respectively. (D, E) Western blotting analyses for CX3CR1 and LC3B (D) or IL‐1β (E) protein levels in astroglial cells (C6) with co‐treatment of CGRP (1 μM) and AA (20 μM) for 4 h. (F, G) Western blot analyses of CX3CR1 and LC3B (F) or IL‐1β (G) expression in the dorsal quadrant of L4‐L5 spinal segment ipsilateral to the operation side on 0, 1, 3, 5, 7, 10, and 14 days after CCI surgery, respectively. (H‐K) Western blot analyses of CX3CR1 or IL‐1β expression in the dorsal quadrant of L4‐L5 spinal segment ipsilateral to the operation side with CCI surgery for 5 and 7 days, respectively. Data were obtained from animals treated with daily intrathecal injection of either 1 μM CGRP (10 μl), 2 μM CGRP8‐37(10 μl), 20 μM AA (10 μl), or vehicle (10 μl) for 4 and 6 days, respectively. The mean optic densities of the proteins were calculated by normalizing to GAPDH. All values are expressed as the means ± SEMs (*n* = 4).*p < 0.05 versus sham groups; #*p* < 0.05 versus CCI only groups

Since H3K9ac was an active chromatin marker and often associated with positive gene expression, we next addressed the impact of gain or loss of H3K9ac induced by CGRP on gene expression. We selected a subset of genes annotated with the GO terms enriched among genes gaining or losing H3K9ac (ATG12, ATG4C, CX3CR1, MTMR14, HMOX1, RET), and assessed their expression in astroglial cells after treatment of CGRP for 4 h by qRT‐PCR. Results showed that most of genes gaining H3K9ac became significantly up‐regulated, and genes losing this mark were significantly down‐regulated compared to controls (Figure 6A).

To further understand the effect of CGRP on astrocytes, candidate gene CX3CR1, proinflammatory cytokine IL‐1β, and autophagic marker LC3B were selected and their protein expressions in astroglial cells were examined following CGRP treatment. Western blot data showed that CGRP evoked significant increases in CX3CR1, LC3B, (Figure 6B) and IL‐1β (Figure 6C) protein expressions in astroglial cells treated with CGRP for 1, 2, 4, 6, and 12 h, respectively. However, CGRP with AA partially or completely blocked the increased effect of CGRP on the expression of CX3CR1, LC3B (Figure 6D), and IL‐1β (Figure 6E) in astroglial cells after 4‐h treatment (*p* < 0.05; *n* = 4).

Furthermore, Western blot results showed that CC significantly increased CX3CR1, LC3B (Figure 6F), and IL‐1β (Figure 6G) protein levels in the dorsal quadrant of L4‐L5 spinal segment ipsilateral to the operation side after CCI injury on postoperative days 1, 3, 5, 7, 10, and 14, respectively (*p* < 0.05; *n* = 4). CGRP treatment also significantly increased CX3CR1 and IL‐1β protein levels in the dorsal quadrant of L4‐L5 spinal segment ipsilateral to the operation side on postoperative days 5 and 7, respectively, compared with sham groups (*p* < 0.05; *n* = 4) (Figure 6H‐K). However, CCI with CGRP8‐37 or AA markedly reversed the CCI‐induced increases of CX3CR1 and IL‐1β protein expressions (*p* < 0.05; *n* = 4).

## 4 DISCUSSION

The present study was to examine the facilitating nociceptive effect and possible mechanism of H3K9ac by CGRP in CCI rats. We found that CGRP was able to up‐regulate H3K9ac expression in astrocytes through HATs. ChIP‐seq data indicated that treatment of CGRP with astroglial cells remarkably altered enrichments of H3K9ac on gene promoters that were mostly associated with proliferation, autophagy, and macrophage chemotaxis. Importantly, spinal inhibition of CGRP and HATs attenuated the development of neuropathic pain in CCI rats, accompanied by suppressed expression of H3K9ac, CX3CR1, and IL‐1β protein levels in the spinal dorsal horn. Our findings highly indicate that CGRP is implicated in the development of neuropathic pain through regulating the activation of astrocytes via H3K9ac in the spinal dorsal horn following nerve injury.

Accumulating evidence implicates a role of CGRP in the spinal mechanisms of the processing of nociceptive information, which involves increased neuron‐glia interactions.^13^, ^29^ The major origin of CGRP in the dorsal spinal cord has been reported to be extrinsic, from afferent fibers which are derived from neurons in the dorsal root ganglia.^30^ Previous study showed that CGRP receptors present in most of the dorsal horn neurons and co‐localize with AMPA receptor.^29^ Functional evidence indicates astrocytes expressed CGRP receptor, which confers selectivity for CGRP and CGRP8‐37.^14^, ^16^ Following nerve injury, the release of CGRP from terminals of sensory neurons in the dorsal horn might not only facilitate glutamate‐driven neuronal nociceptive signaling, but also act on astrocytic CGRP receptors and lead to astrocyte activation.^13^, ^17^, ^30^ The activated astrocytes can produce a repertoire of proinflammatory mediators, such as IL‐8, MCP‐1, and NO.^30^ Therefore, astrocytic release of cytokines and chemokines can further exacerbate the pathological processes of degenerative or inflammatory CNS diseases.^31^ In the present work, we showed that CGRP‐immunoreactive levels were significantly correlated with GFAP expression in the dorsal horn after CCI surgery. Importantly, most CGRP‐immunostained fibers were found to closely approach GFAP‐immunopositive astrocytes. These data suggest that spinal astrocytes may be activated by CGRP released from CGRP containing fibers after CCI, which is increased in CGRP‐positive terminals. Therefore, CGRP release from afferent terminals is critically involved in the initiation and maintenance of astrocyte activation in spinal dorsal horn.^13^, ^29^

H3K9ac signals are increased in a group of genes that are up‐regulated in glial cells and involved in gliogenesis.^32^, ^33^ The exact mechanism by which CGRP stimulates H3K9ac in astrocyte remains unclear. Cheng et al. found that the CBP/p300 is required for GFAP expression by acetylating H3K9 and H3K14 during astrocyte differentiation in development.^19^ CGRP signaling through its receptor activates a pathway involving cAMP/PKA, which promotes recruitment of the histone acetyltransferase CBP/p300 that mediate histone H3K9 acetylation.^34^, ^35^ Therefore, the up‐regulation of H3K9ac by CGRP might be mediated by CBP/p300 via cAMP/PKA signal pathway and that this links to the increase of GFAP and activation of astrocytes. In the present study, we found that CGRP significantly increased the expression of H3K9ac in spinal astrocytes. Moreover, CGRP antagonist and HAT inhibitor suppressed CCI‐induced H3K9ac expression, which were concurrently associated with the attenuated mechanical and thermal hyperalgesia. Thus, in the spinal cord, the increased release of sensory neuron‐derived CGRP may activate CGRP receptors expressed on astrocytes leading to up‐regulation of H3K9ac of which can mediate inflammatory gene expression, thereby facilitating nociception in CCI rats.^32^, ^33^

In order to obtain insights into H3K9ac target gene function, GO and KEGG pathway analyses were applied to the H3K9ac target gene pool. Bioinformatics analyses showed that genes gaining H3K9ac were mainly associated with autophagy and macrophage chemotaxis. Autophagy plays a critical role in maintaining astrocytic functions and up‐regulation of autophagy reduced cell apoptosis in cultured astrocytes subjected to oxygen and glucose deprivation.^36^, ^37^ Several reports showed that autophagy was mostly related to allodynia, hyperalgesia, and astrocyte activation in a rat model of neuropathic pain,^38^, ^39^ and we demonstrated that CGRP increased the autophagy marker LC3B expression in cultured astrocytes. Macrophage chemotaxis is linked to activated astrocytes, contributes to central sensitization, and maintains chronic and neuropathic pain.^40^ During neuroinflammation, astrocyte can induce chemotaxis of macrophages/microglia to sites of injury/inflammation and induce cytokine and chemokine expression.^41^ Furthermore, genes losing H3K9ac were mainly associated with suppression of gene transcription and promotion of cell apoptosis, consistent with our results that CGRP promoted astrocyte proliferation through HATs. Therefore, association with autophagy‐, proliferation‐, and inflammation‐related genes seems, therefore, to be a feature of CGRP‐mediated H3K9ac enrichment. On the other hand, we demonstrated that CGRP promoted the proliferation of astroglial cells and AA completely blocked the increased effect of CGRP on cell proliferation. Moreover, AA alone robustly inhibited the cell proliferation. Consistent with our result, previous reports showed that AA suppresses cell survival, proliferation, invasion, and inflammation through modulation of NF‐kB, androgen receptor, and p53 signal pathways,^42^, ^43^ suggesting that AA functions in diverse cellular pathways and many are involved in cell proliferation.

Among identified candidate genes gaining H3K9ac, ATG12, ATG4C, and MTMR14 are autophagy‐related genes and play a promoting role in autophagy in glial cells.^44^, ^45^, ^46^ Previous study showed that ATG12 promoted astrocyte activation and the expression of the inflammatory mediators, such as iNOS and COX‐2 through activation of TLR4 signaling.^44^ Furthermore, among candidate genes losing H3K9ac, HMOX1, and RET are crucial for the protection of astrocytes and neurons from toxicity in CNS.^47^, ^48^ Our results showed that CGRP could increase H3K9ac enrichment on genes of ATG12, ATG4C, MTMR14, and attenuate this mark on genes of HMOX1 as well as RET, these might contribute to astrocytes proliferation, activation, autophagy, and production of proinflammatory mediators by the alteration of this active mark H3K9ac in astrocytes.

Spinal astrocytes are the main source of the IL‐1β which, in turn, acts on its neuronal and astrocytic IL‐1 receptor (IL‐1R) leading to enhanced activation of the local cells (neurons and glia as well) and can lead to the prolonged maintenance of chronic pain.^49^ Despite CX3CR1 is mainly expressed by activated microglia, spinal activated‐astrocytes also express CX3CR1 following injury,^50^ consistent with our results that CX3CR1 was increased in cultured astrocytes by HATs following CGRP treatment. Our data also strongly indicated that pharmacological suppression of the increased activities of HATs and CGRP attenuate the development of neuropathic pain via suppressing induction of H3K9ac and production of CX3CR1 and IL‐1β. Remarkably, intrathecal injection of CGRP and CCI resulted in a tremendous increase in the number of astrocytes with H3K9ac expression. Given that astrocytic activation induced by CGRP, as evident by an increased GFAP expression, was found at the same time, it is conceivable that the increased expression of H3K9ac in astrocytes contributes importantly to the astrocytic activation and its production of inflammatory mediators,^49^, ^50^ which ultimately brings about neuroinflammation in the spinal dorsal horn. In line with our results, recent studies have indicated that several pro‐inflammatory cytokines and chemokines (IL‐1, CXCL12/CXCR4, etc.) may also function as mediators of neuropathic pain.^51^, ^52^, ^53^ For example, the expression of CXCL12 mainly increased in neurons while the expression of CXCR4 was increased both in astrocytes and neurons in the spinal dorsal horn after nerve injury.^51^ Treatment with IL‐1R antagonist (IL‐1RA) has demonstrated the potential to induce analgesia in a neuropathic pain model.^52^ Additionally, reactive astrocytes can directly influence neuronal activity through the release of various transmitters (e.g., GABA), acting on membrane receptors expressed by neurons.^54^, ^55^ Previous studies have also shown that chronic pain is correlated with the appearance of reactive astrocytes in the spinal dorsal horn and that reactive astrocytes release more GABA,^56^, ^57^ suggesting the possibility that GABA released from astrocytes may play a role in the processing of nociceptive information. However, further research is still needed.

Although C6 astroglial cells partially display astrocyte features, this C6 cell line is widely used as an astrocyte‐like cell line to study astrocytic function, for example, glutamate uptake, glutamine synthetase activity, S100B secretion, and parameters of oxidative stress.^58^ Moreover, this cell line responds quickly to external stimuli, such as H_2_O_2_, which can generate oxidative‐nitrosative stress.^59^ Previous reports showed that elevation in the level of intracellular cAMP induced astrocytic differentiation of C6 cells by increased GFAP levels and hypertrophic changes.^60^, ^61^ C6 cells express CGRP receptors that couple the receptor to the cellular signal pathway leading to increased intracellular cAMP.^62^ Therefore, treatment of CGRP with C6 cells might promote astrocytic differentiation and maturation of C6 cells.

In conclusion, our current study reveals that CGRP plays a critical role in the development of neuropathic pain through regulating neuroinflammation via H3K9ac in astrocytes. Genomic analyses suggested that genes with enrichments of H3K9ac induced by CGRP are involved in astrocytic proliferation‐, autophagy‐, and inflammation‐related gene expression that might be associated with neuropathic pain. CX3CR1 and IL‐1β, key mediators of astrocyte activation, were identified in CCI rat model and might be crucial in the development of neuropathic pain. These findings suggest that CGRP receptors in astrocytes may act as an important therapeutic target for neuropathic pain by suppressing the neuron‐glia interaction. However, further studies are needed to confirm our results.

## CONFLICT OF INTEREST

The authors declare no conflict of interest.

## Supporting information

Figure S1

Table S1

Table S2

Table S3

Table S4

Figure Legends

## Data Availability

The data that support the findings of this study are available from the corresponding author upon reasonable request.
