# Supplementary figures and images for: Calcitonin gene‐related peptide induces the histone H3 lysine 9 acetylation in astrocytes associated with neuroinflammation in rats with neuropathic pain
Source: CNS Neurosci Ther. 2021 Aug 16;27(11):1409–24. doi: 10.1111/cns.13720 (PMC8504526; doi:10.1111/cns.13720)

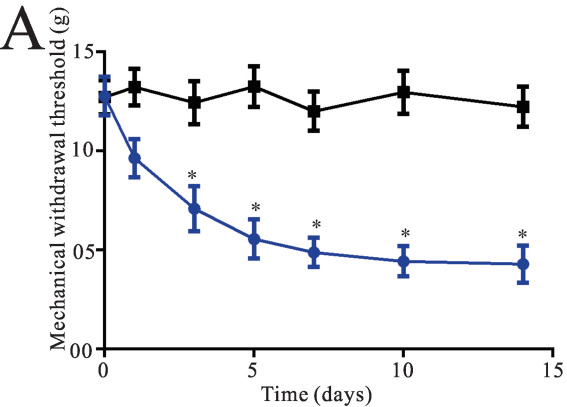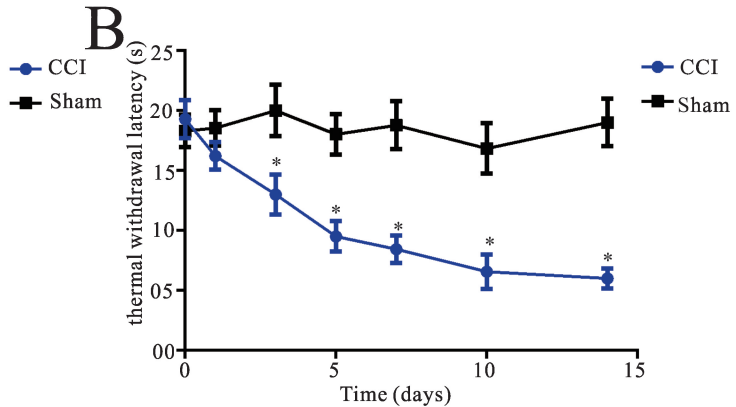

Supplement: Supplementary file 1 — Figure S1 [file CNS-27-1409-s005.pdf]
