# Supplementary material for: Calcitonin gene‐related peptide induces the histone H3 lysine 9 acetylation in astrocytes associated with neuroinflammation in rats with neuropathic pain
Source: CNS Neurosci Ther. 2021 Aug 16;27(11):1409–24. doi: 10.1111/cns.13720 (PMC8504526; doi:10.1111/cns.13720)

Full unedited blots for Figure 1C

CGRP

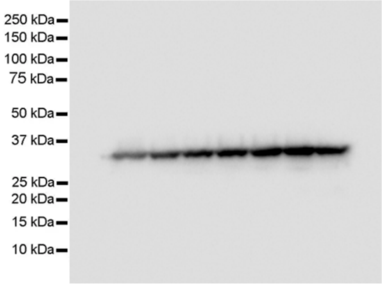

GFAP

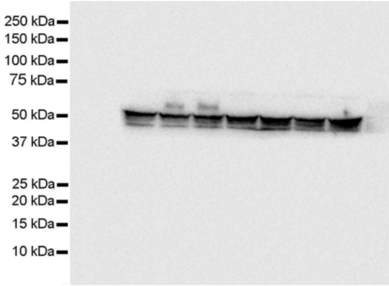

GAPDH

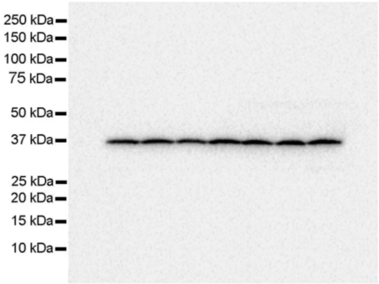

Full unedited blots for Figure 2C

H3K9ac

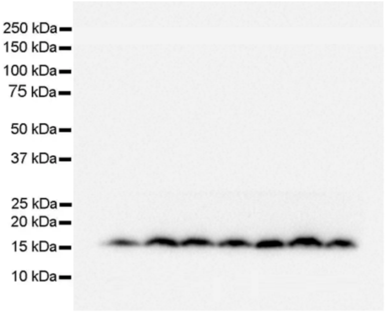

GAPDH

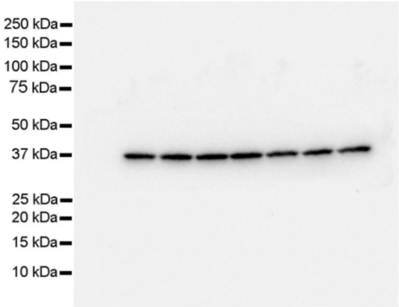

Full unedited blots for Figure 3B

H3K9ac

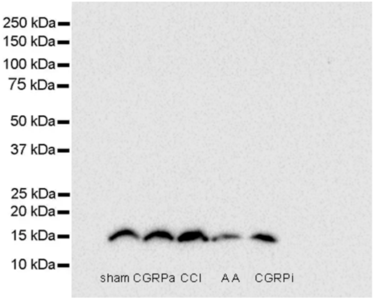

C

H3K9ac

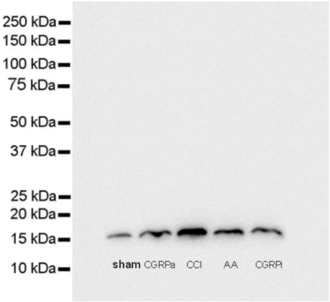

D

H3K9ac

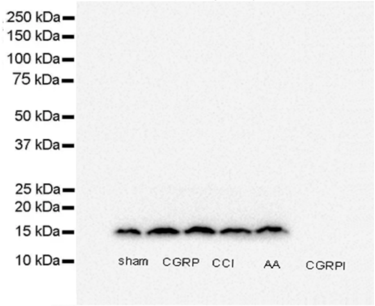

E

H3K9ac

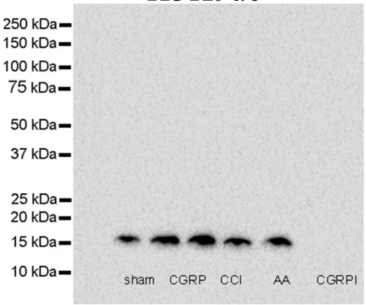

GAPDH

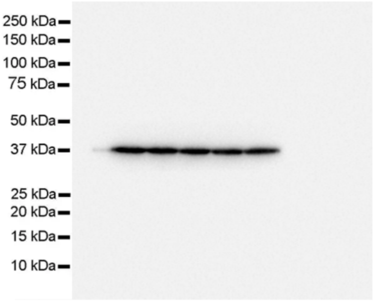

GAPDH

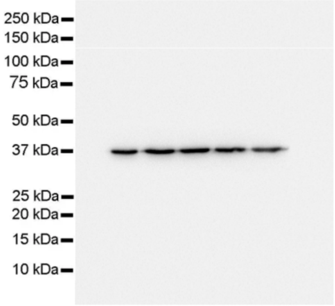

GAPDH

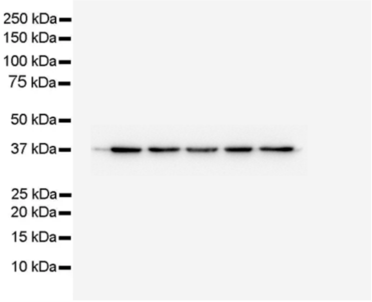

GAPDH

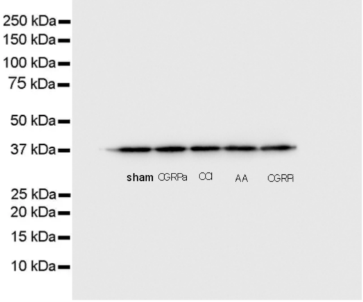

Full unedited blots for Figure 4B

H3K9ac

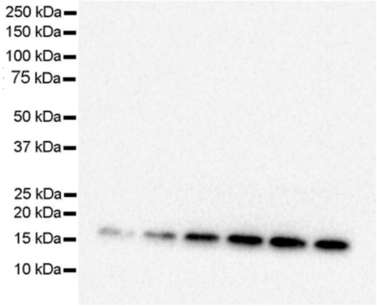

C

H3K9ac

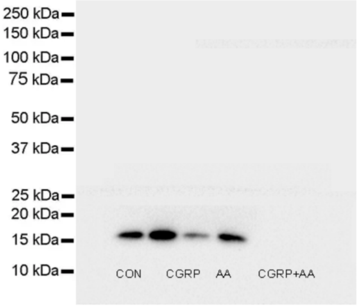

D

GFAP

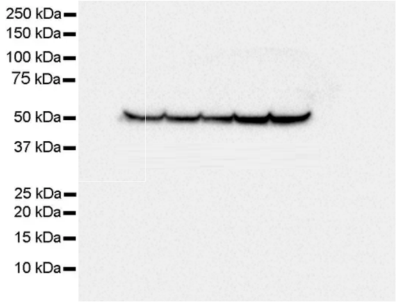

GAPDH

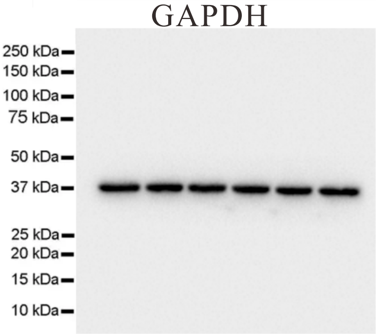

GAPDH

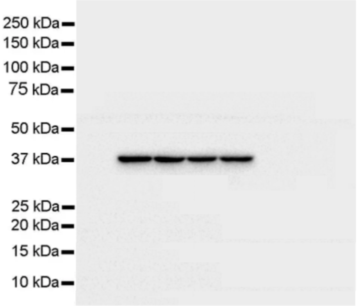

GAPDH

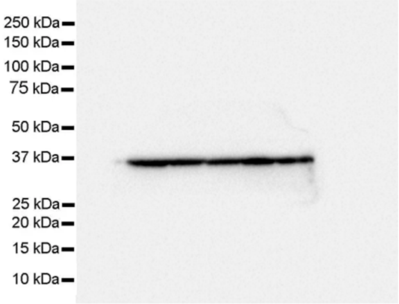

Full unedited blots for Figure 6B

LC3B

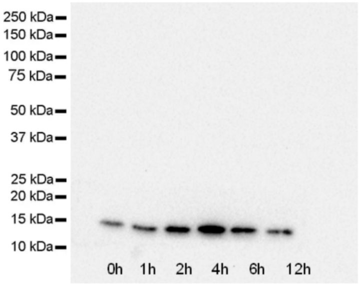

CX3CR1

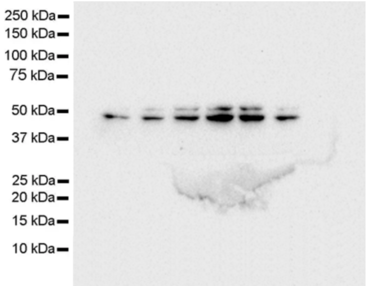

GAPDH

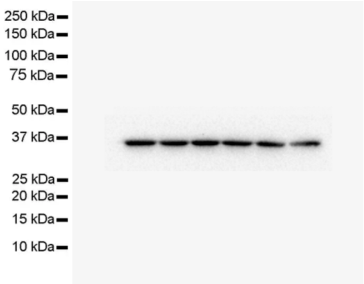

Full unedited blots for Figure 6C

IL-1β

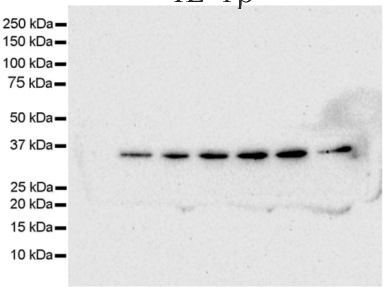

GAPDH

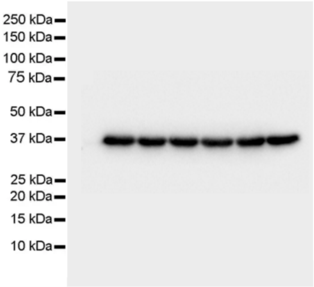

Full unedited blots for Figure 6D

LC3B

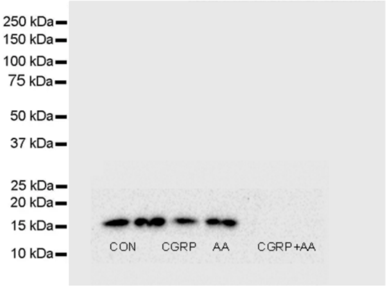

CX3CR1

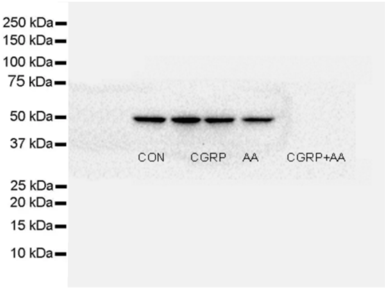

GAPDH

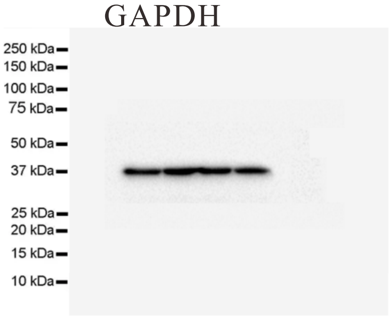

Full unedited blots for Figure 6E

IL-1β

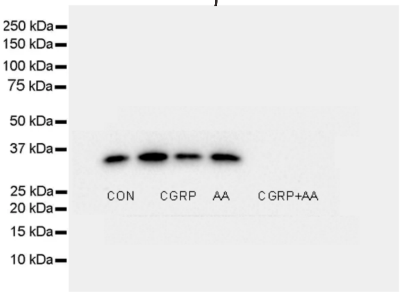

GAPDH

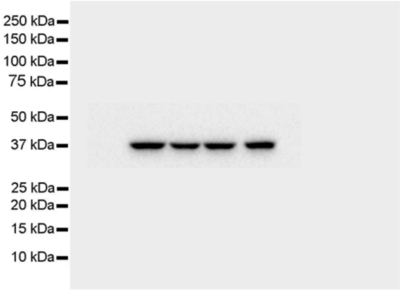

Full unedited blots for Figure 6F

LC3B

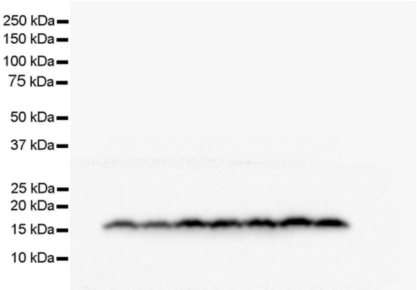

CX3CR1

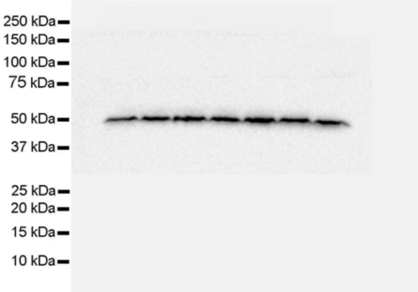

GAPDH

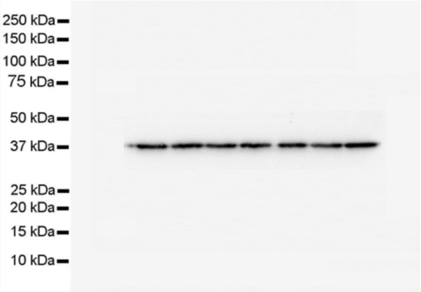

Full unedited blots for Figure 6G

IL-1β

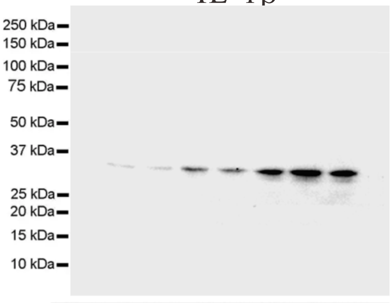

GAPDH

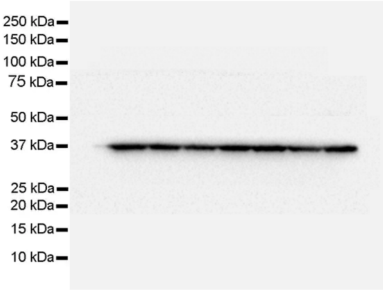

Full unedited blots for Figure 6H

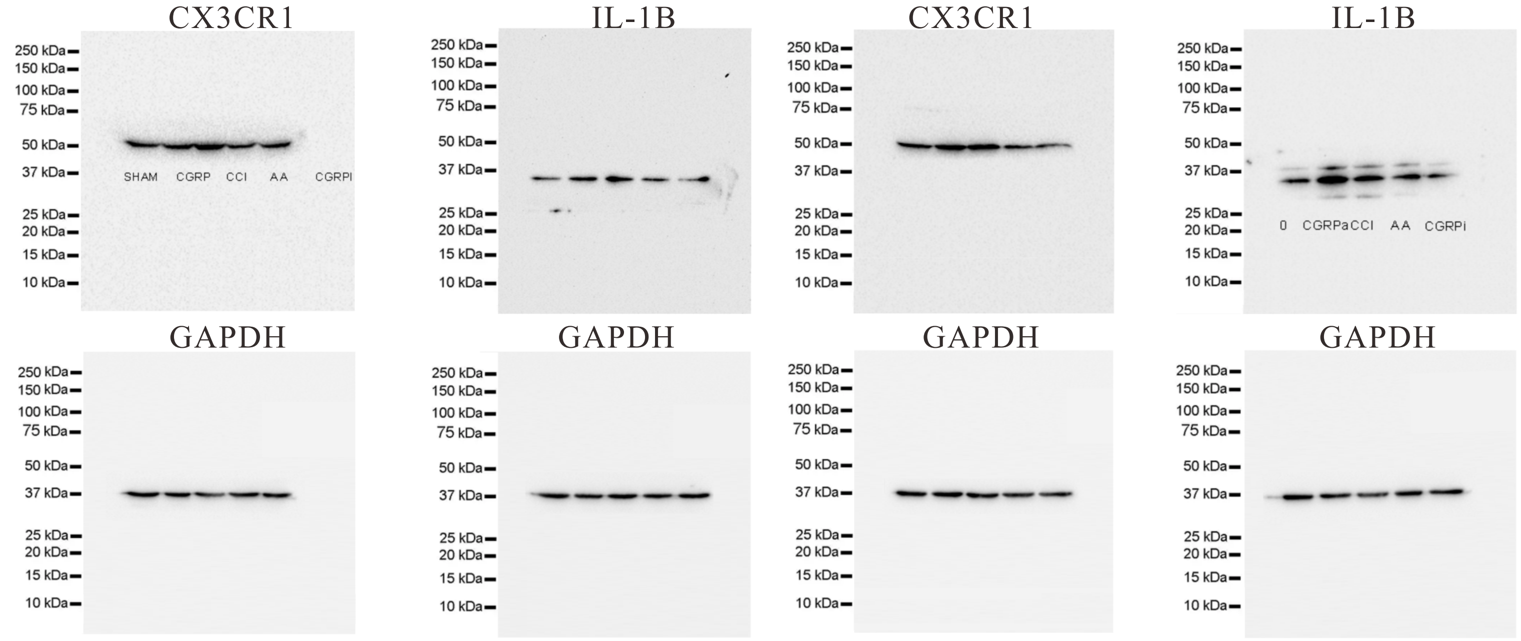

Supplement: Supplementary file 6 — Figure Legends [file CNS-27-1409-s001.pdf]
